# Glymphatic Function as a Prognostic Biomarker in Prolonged Disorders of Consciousness

**DOI:** 10.1111/cns.70526

**Published:** 2025-07-23

**Authors:** Dian‐Wei Wu, Chang‐Geng Song, Rong Chen, Jing‐Jing Zhao, Ying‐Chi Zhang, Xuan Wang, Zhong‐Qing Sun, Xiao‐Gang Kang, Qiong Gao, Wen Jiang

**Affiliations:** ^1^ Department of Neurology, Xijing Hospital Fourth Military Medical University Xi'an China

**Keywords:** BOLD‐CSF coupling, consciousness recovery, glymphatic function, prognosis, prolonged disorders of consciousness

## Abstract

**Objective:**

The glymphatic system is a major waste clearance system in the central nervous system. We aim to investigate the glymphatic function and its prognostic values in patients with prolonged disorders of consciousness (pDoC).

**Methods:**

We conducted a prospective and explorative cohort study including 40 patients with pDoC and 20 healthy controls. Glymphatic function was measured with the global and regional blood‐oxygen‐level‐dependent and cerebrospinal fluid (BOLD‐CSF) couplings, characterized by time‐lags and strengths of the couplings. The clinical outcome was defined as improvement and no improvement in consciousness 6 months after enrollment, determined via a structured telephone follow‐up based on the Coma Recovery Scale‐Revised (CRS‐R) score.

**Results:**

Patients with pDoC exhibited significantly delayed time‐lags in BOLD‐CSF coupling (*p* < 0.05) and significantly reduced coupling strengths (*p <* 0.05) when compared to healthy controls. Follow‐up studies indicated that shorter global BOLD‐CSF coupling time‐lags can predict an improved consciousness 6 months after enrollment, with an area under the receiver operating characteristic curve of 0.837, a sensitivity of 82.4%, and an accuracy of 85.7% using a cutoff point of 7.5.

**Conclusion:**

The glymphatic system was impaired in patients with pDoC, and its function, measured by BOLD‐CSF coupling, can serve as a novel prognostic biomarker.

## Graphical abstract

This study examines glymphatic dysfunction in pDoC via BOLD‐CSF coupling analysis using MRI. pDoC patients show delayed time‐lags and reduced coupling strengths, with time‐lags predicting consciousness recovery. Findings suggest glymphatic dysfunction as a potential biomarker for consciousness recovery in pDoC.

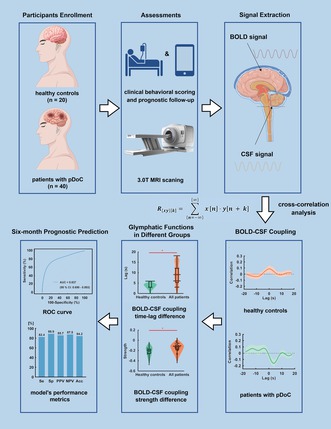

## 1 Introduction

Prolonged disorders of consciousness (pDoC) refer to conditions characterized by severe impairment of consciousness lasting beyond 28 days, including vegetative state/unresponsive wakefulness syndrome (VS/UWS) and minimally conscious state (MCS) [1]. pDoC typically occurs following severe brain injuries such as anoxia, traumatic brain injury (TBI), intracerebral hemorrhage, and ischemic stroke, and its prolonged course imposes a significant burden on patients, families, and society, consuming substantial healthcare resources [2, 3]. Accurate prognostic prediction of consciousness recovery in pDoC is essential for guiding clinical decision‐making and optimizing treatment strategies. However, current prognostic tools, including electroencephalography, magnetic resonance imaging (MRI), and behavioral evaluations, fall short of meeting the needs for precise prognostic evaluation. Therefore, there is an urgent need for new predictive approaches [4, 5, 6].

The glymphatic system is a crucial component of the waste clearance process in the central nervous system [7, 8]. It allows cerebrospinal fluid (CSF) to enter the brain parenchyma through the periarterial space, mix with interstitial fluid, and then drain out through perivenous pathways, carrying waste solutes along [9]. Following acute brain injury, neuronal death and neuroinflammation occur within the brain, leading to the accumulation of metabolic waste and toxic substances [10, 11, 12, 13, 14, 15]. This damages consciousness and adversely affects the recovery of consciousness. The glymphatic system plays a crucial role in clearing metabolic waste and toxic substances in the brains of patients with pDoC. However, there are currently no studies on glymphatic function in pDoC patients.

The coupling between blood‐oxygen‐level‐dependent (BOLD) signals and the CSF signals, measured with functional MRI (fMRI), is a newly introduced indicator of glymphatic function. In their pioneering study, Kiviniemi et al. found that the hemodynamic oscillations induced by neural activity were coupled to CSF flow, and this BOLD‐CSF coupling serves as a proxy for the interplay between cerebral neural activity and glymphatic CSF flow, reflecting how effectively neural activity facilitates CSF movement [16, 17, 18, 19, 20]. Compared with traditional indicators of glymphatic function, BOLD‐CSF coupling more accurately reflects the interplay between neural activity and glymphatic function. In this study, we aimed to investigate the glymphatic function in patients with pDoC via BOLD‐CSF coupling and its prognostic values in consciousness recovery. We hypothesized that better glymphatic function signifies more efficient waste clearance, which lays the foundation for improved recovery of consciousness.

## 2 Methods

### 2.1 Study Setting and Participants

This prospective and explorative cohort study was conducted at the Coma Awakening Center, Department of Neurology, Xijing Hospital, Xi'an, China. Patients and healthy controls were enrolled in a 2:1 ratio between May 2022 and February 2024. The inclusion criteria were: (1) age > 18 years; (2) diagnosis of VS/UWS or MCS upon admission [21]; (3) time since onset > 28 days [22, 23, 24]; and (4) availability of 3T resting‐state fMRI (rs‐fMRI) and T1‐weighted MRI scans. Exclusion criteria included: (1) patients with skull defects; (2) presence of major/unstable medical issues; and (3) exhibiting excessive head motion (greater than 3 mm or 3°) during the rs‐fMRI scan. Age‐ and gender‐matched healthy controls without any pre‐existing diseases or ongoing treatments were recruited. The study adhered to the principles outlined in the Declaration of Helsinki and was approved by the Ethics Committee of Xijing Hospital (XJ‐20222068‐F1). Written informed consents were obtained from the healthy controls and the legal representatives of the patients.

### 2.2 Behavioral Assessment

Two qualified and independent neurologists who were blinded to the outcomes evaluated the level of consciousness using the Coma Recovery Scale‐Revised (CRS‐R) scores [21]. Diagnoses of VS/UWS and MCS (minus or plus) were determined based on the presence of specific CRS‐R subscale items. The baseline consciousness was determined by the highest CRS‐R score from at least two assessments at enrollment.

### 2.3 MRI Acquisition and Preprocessing

Both healthy controls and patients with pDoC underwent a single imaging session after enrollment. For the healthy controls, T1 and rs‐fMRI data were sequentially acquired using a 3.0T MRI scanner (uMR770, United Imaging Healthcare, Shanghai, China). All patients with pDoC underwent a comprehensive multimodal brain MRI scan employing a 3.0T time‐of‐flight positron emission tomography (PET)/MRI scanner (SIGNA PET/MR, GE Healthcare, WI, USA). The acquisition parameters for T1‐weighted structural imaging were standardized across all participants, with a repetition time (TR) of 8.00 ms, echo time (TE) of 3.06 ms, flip angle of 8°, and slice thickness of 1.0 mm. For rs‐fMRI, the parameters were set to TR = 3000 ms, TE = 35 ms, flip angle = 90°, slice thickness = 5 mm, and either 240 slices for healthy controls or 128 slices for all patients, accommodating variations in scan duration due to patient‐specific constraints. To minimize inter‐batch variability stemming from the acquisition of MRI scans in different batches for both patient and control groups, a uniform MRI acquisition sequence was adopted, ensuring consistency in image data quality [25]. All MRI datasets were then visually inspected by two independent investigators who were blinded to the clinical data.

The rs‐fMRI data were preprocessed using DPABI software (version 5.4) [26]. Preprocessing steps involved an initial step of discarding the first 10 images to mitigate instability effects, followed by skull stripping, slice timing correction, realignment, normalization to a standard Montreal Neurological Institute space, and application of spatial Gaussian kernel smoothing with a 4 mm full‐width at half‐maximum.

A high‐pass filter with a frequency range of 0.01–0.1 Hz was applied to remove low‐frequency noise, while linear and quadratic detrending were implemented to address signal drifts. Notably, motion correction was omitted to preserve the integrity of CSF signal data, as previously justified [17]. Additionally, during preprocessing, the number of time slices for healthy controls was adjusted to match that of the pDoC patients, ensuring comparability between the two datasets.

### 2.4 BOLD‐CSF Coupling

**FIGURE 1:**
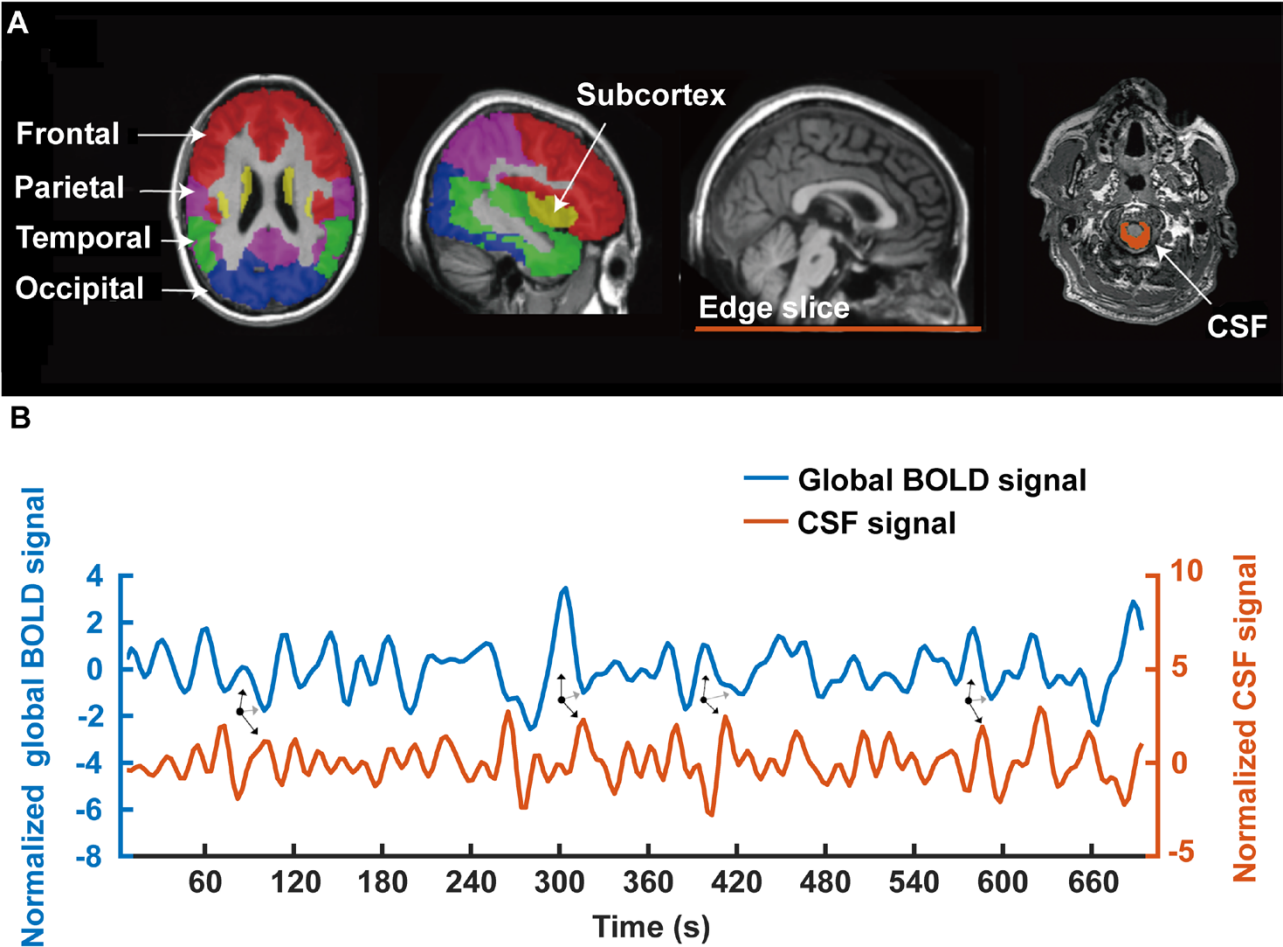
The coupling between BOLD and CSF signals. (A) The global BOLD signals were extracted from the gray matter region of the cerebrum and subdivided into five parts: Frontal, parietal, temporal, occipital, and subcortex regions. The CSF signals were obtained from the edge slice of fMRI between the upper spinal cord/medulla oblongata and the lower cerebellum. (B) The coupling between global BOLD and CSF signals in a representative healthy control. Notably, a positive BOLD signal peak (upward black arrows) typically preceeds a positive CSF signal peak (downward black arrows) in temporal sequence, and is subsequently succeeded by a negative BOLD signal peak (gray arrows). BOLD, blood‐oxygen‐level‐dependent; CSF, cerebrospinal fluid; fMRI, functional magnetic resonance imaging.

Glymphatic system function was assessed through BOLD‐CSF coupling analysis following established protocols [17, 18]. The rs‐fMRI data were utilized to extract both CSF and cerebral BOLD signals. CSF signals were defined as the average BOLD signal within a region of interest (ROI) encompassing the CSF space located between the medulla oblongata and the inferior cerebellum. These signals were extracted from the edge slices of each participant's rs‐fMRI scans to maximize sensitivity to inflow effects [17, 18, 27, 28]. During ROI delineation, potential blood vessels were meticulously excluded to prevent contamination of the CSF signal (Figure 1A). To obtain cerebral BOLD signals, the global cerebrum was first identified and subsequently segmented into frontal, parietal, temporal, occipital, and subcortex regions using the Automated Anatomical Labeling Atlas 3 (AAL3) (Figure 1A) [29]. From this segmentation, global cerebral BOLD (global BOLD) signals and regional BOLD signals corresponding to the aforementioned subregions were extracted.

A representative example of the CSF signal and the mean global BOLD from a healthy control is shown in Figure 1B, demonstrating a correlation between the CSF signal and mean global BOLD, with the BOLD signal peak often preceding the CSF signal peak across the entire cerebrum. The BOLD signal reflects extensive cortical activity, which triggers local neurovascular responses, leading to changes in cerebral blood flow and volume. These changes, in turn, drive CSF flow and facilitate waste clearance from the brain [20].

To assess the glymphatic system function, BOLD‐CSF coupling was quantified by calculating the cross‐correlation coefficients between CSF signals and both global BOLD signals as well as regional BOLD signals across varying time lags (ranging from −18 to 18 s). Two indicators were employed to characterize the BOLD‐CSF coupling: (1) BOLD‐CSF time lag, defined as the post‐zero latency of maximum negative correlation, reflecting CSF penetration dynamics [30]; (2) Strength of BOLD‐CSF coupling: calculated as the value of the post‐zero maximal negative cross‐correlation coefficient, with higher absolute values indicating enhanced glymphatic system efficiency [28].

### 2.5 Definition of Outcomes

The clinical outcome was independently assessed 6 months after enrollment by a trained neurologist, who was blinded to the clinical data, via a structured telephone interview based on the CRS‐R score [31, 32, 33]. Patients were categorized into the improved consciousness group if they transitioned from VS/UWS to MCS or to emergence from MCS (EMCS), from MCS minus to MCS plus or to EMCS, and from MCS plus to EMCS. Patients with unimproved consciousness were defined as those with a reduced or unchanged level of consciousness.

### 2.6 Statistical Analysis

Continuous variables were expressed as mean ± standard deviation (SD) or median (interquartile range, IQR), and categorical variables were expressed as percentages. Normality of continuous variables was assessed using the Shapiro–Wilk test. Continuous variables were analyzed using Student's *t*‐test or one‐way ANOVA for normal distribution, and the Mann‐Whitney *U* test or the Kruskal‐Wallis *H* test for skewed distribution. Categorical variables were analyzed using the *χ* ^2^ test. Based on the results of normality testing, either Spearman's rank‐order correlation or Pearson's correlation coefficient was used to analyze the relationships between clinical demographic data and BOLD‐CSF coupling indicators, as appropriate. The false discovery rate (FDR) correction was applied when comparing coupling parameters (time‐lag/strength) across brain regions between groups [34]. To evaluate the statistical significance of the BOLD‐CSF correlation within specific groups, we randomly paired BOLD and CSF signals from individual participants and computed the corresponding correlations. This permutation test was repeated 1000 times to ensure the reliability of our results.

To assess the association between BOLD‐CSF coupling and patients' prognosis, we implemented a bidirectional stepwise logistic regression, specifically designed to minimize potential multicollinearity among variables, to identify the BOLD‐CSF coupling features most relevant to the 6‐month prognosis of patients with pDoC [35]. The receiver operating characteristic curve (ROC), sensitivity, specificity, positive predictive value (PPV), negative predictive value (NPV), and the maximum accuracy determined by the cut‐off value (Youden Index) were used to determine the predictive capability of the BOLD‐CSF coupling indicators. Predictive accuracy was calculated as the average of sensitivity and specificity. Statistical significance was defined as a *p*‐value < 0.05. Statistical analyses were performed using MATLAB (version 2022a), GraphPad Prism 9.0, and R Version 4.3.1.

## 3 Results

### 3.1 Demographic, Clinical Characteristics, and Outcomes of the Study Population

A total of 40 patients with pDoC (24 males and 16 females, mean age 47.55 ± 13.65 years) and 20 healthy controls (13 males and 7 females, mean age 41.90 ± 9.87 years) were recruited. The detailed demographic and clinical characteristics of the individuals are presented in Table S1. There were no significant differences in age (*p* = 0.105) or gender (*p* = 0.707) between healthy controls and the pDoC patients. The mean age of the patients was 47.55 ± 13.65 (mean ± SD) years, and 60.0% of them were male. The median (IQR) CRS‐R score of the pDoC patients was 7.00 (5.00, 10.00), and 55% of the patients were VS/UWS. The time since injury ranged from 33 to 76 days, with a median of 57 days. The most common etiology of pDoC was anoxia (50%), followed by TBI (15%), hemorrhagic stroke (15%), ischemic stroke (10%), and metabolic encephalopathy (10%). At 6 months post‐enrollment, no cases were lost to follow‐up. Among the patients, 19 (47.5%) achieved improved consciousness, whereas 21 (52.5%) showed no improvement. There were no significant differences in baseline clinical characteristics between the two groups (Table 1).

**TABLE 1:** Demographic, clinical, and BOLD‐CSF coupling characteristics of patients with improved and unimproved consciousness.

Variable	Total (*n* = 40)	Improved consciousness (*n* = 19)	Unimproved consciousness (*n* = 21)	*p*
Age, mean ± SD, years	47.55 ± 13.65	47.05 ± 12.38	48.00 ± 14.99	0.830
Gender: male, No. (%)	24 (60.00)	13 (61.90)	11 (57.89)	0.796
Baseline CRS‐R, median (IQR)	7.00 (5.00, 10.00)	8.00 (6.00, 10.00)	6.00 (4.00, 12.00)	0.414
Baseline consciousness state				0.344
VS/UWS, No. (%)	22 (55.00)	9 (47.37)	13 (61.90)	
MCS‐, No. (%)	12 (30.00)	8 (42.11)	4 (19.05)	
MCS+, No. (%)	6 (15.00)	2 (10.53)	4 (19.05)	
Time since injury, median (IQR), days	56.00 (33.00, 76.00)	57.00 (37.00, 77.00)	49.00 (34.00, 77.00)	0.957
Etiology				0.146
Anoxia, No. (%)	20 (50.00)	6 (31.58)	14 (66.67)	
TBI, No. (%)	6 (15.00)	4 (21.05)	2 (9.52)	
Stroke, No. (%)	10 (25.00)	7 (36.84)	3 (14.29)	
Others, No. (%)	4 (10.00)	2 (10.53)	2 (9.52)	
BOLD‐CSF coupling time‐lags, median (IQR), seconds				
Global	9.00 (5.25, 12.00)	6.00 (3.00, 6.00)	9.00 (9.00, 14.25)	**< 0.001**
Frontal	7.50 (3.00, 15.00)	6.00 (3.00, 12.00)	9.00 (6.75, 14.25)	**0.030** ^†^
Parietal	7.50 (3.00, 12.75)	3.00 (3.00, 6.00)	10.50 (6.75, 17.25)	**0.003** ^†^
Occipital	9.00 (3.00, 12.75)	3.00 (0.00, 6.00)	10.50 (6.75, 15.00)	**0.005** ^†^
Temporal	9.00 (3.00, 15.00)	3.00 (0.00, 9.00)	12.00 (9.00, 15.00)	**0.018** ^†^
Subcortex	6.00 (3.00, 12.00)	6.00 (0.00, 12.00)	7.50 (6.00, 12.00)	0.291^†^
BOLD‐CSF coupling strengths, mean ± SD				
Global	−0.16 ± 0.11	−0.15 ± 0.10	−0.16 ± 0.12	0.797
Frontal	−0.14 ± 0.11	−0.12 ± 0.10	−0.15 ± 0.11	0.475
Parietal	−0.16 ± 0.11	−0.15 ± 0.12	−0.17 ± 0.10	0.562
Occipital	−0.17 ± 0.12	−0.16 ± 0.13	−0.17 ± 0.12	0.832
Temporal	−0.15 ± 0.11	−0.14 ± 0.08	−0.17 ± 0.14	0.486
Subcortex	−0.20 ± 0.11	−0.22 ± 0.10	−0.17 ± 0.12	0.227

*Note:* Time since injury: the interval between brain injury onset and MRI acquisition. Statistically significant effects are marked in bold (*p* < 0.05).

Abbreviations: BOLD‐CSF coupling, the coupling between blood‐oxygen‐level‐dependent signals and cerebrospinal fluid signals; CRS‐R, coma recovery scale‐revised; IQR, interquartile range; MCS, minimally conscious state; SD, standard deviation; TBI, traumatic brain injury; VS/UWS, vegetative state/unresponsive wakefulness syndrome.

^†^ *p*‐value after false discovery rate correction.

### 3.2 BOLD‐CSF Coupling in Healthy Controls and pDoC Patients

**FIGURE 2:**
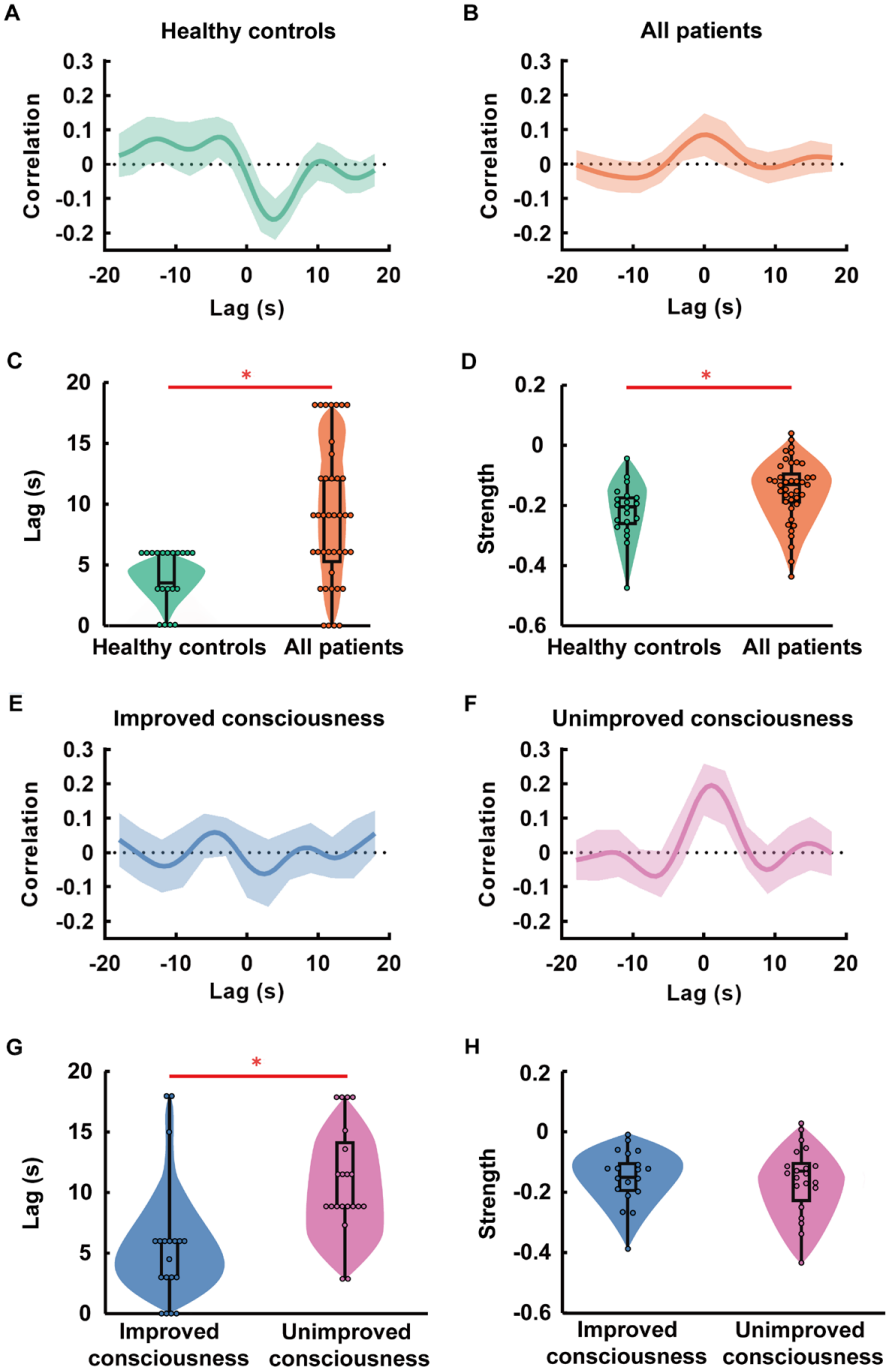
BOLD‐CSF coupling in healthy controls and patients with pDoC. (A) Mean global BOLD‐CSF coupling in the healthy control group. (B) Mean global BOLD‐CSF coupling in all patients with pDoC. (C) Comparisons of global BOLD‐CSF coupling time‐lags in healthy controls and all patients with pDoC. (D) Comparisons of global BOLD‐CSF coupling strengths in healthy controls and all patients with pDoC. (E) Mean global BOLD‐CSF coupling in patients with improved consciousness. (F) Mean global BOLD‐CSF coupling in patients with unimproved consciousness. (G) Comparisons of global BOLD‐CSF coupling time‐lags in patients with improved consciousness and unimproved consciousness. (H) Comparisons of global BOLD‐CSF coupling strengths in patients with improved consciousness and unimproved consciousness. *p < 0.05. BOLD‐CSF coupling, the coupling between blood‐oxygen‐level‐dependent signals and cerebrospinal fluid signals; pDoC, prolonged disorders of consciousness.

Healthy controls exhibited a positive peak in mean BOLD‐CSF coupling at a time‐lag of −2 to −3 s (*r* range: 0.02 to 0.13, *p* < 0.001, permutation test) and a negative peak at a time‐lag of 3 to 4 s (*r* range: −0.21 to −0.09, *p* < 0.001, permutation test) (Figure 2A). In contrast, patients with pDoC displayed a positive peak at a lag of 0 s (*r* range: 0.02 to 0.14, *p* < 0.001, permutation test) and a non‐significant negative peak at a time‐lag of 9 s (*r* range: −0.06 to 0.04, *p* = 0.848, permutation test) (Figure 2B). We further quantified the BOLD‐CSF coupling time‐lag and strength. Patients with pDoC exhibited a significantly increased global BOLD‐CSF coupling time‐lag compared to healthy controls (median [IQR]: 9.00 [5.25, 12.00] vs. 6.00 [3.00, 6.00], *p* = 0.0008, Mann–Whitney *U* test) (Figure 2C). This increase was also observed in all regional BOLD‐CSF coupling time‐lags, including the frontal (9.00 [3.00, 15.00] vs. 3.50 [3.00, 6.00], adjusted *p* = 0.0058, FDR‐corrected), parietal (7.50 [3.00, 12.75] vs. 6.00 [3.00, 6.00], adjusted *p* = 0.0111), occipital (9.00 [3.00, 12.75] vs. 3.00 [3.00, 6.00], adjusted *p* = 0.0058, Mann–Whitney *U* test, FDR‐corrected), temporal (9.00 [3.00, 15.00] vs. 3.00 [2.25, 6.00], adjusted *p* = 0.0030), and subcortex regions (6.00 [3.00, 12.00] vs. 4.50 [2.25, 6.00], adjusted *p* = 0.0145, Mann–Whitney *U* test, FDR‐corrected) (Figure S1A). Additionally, patients with pDoC demonstrated significantly weaker global BOLD‐CSF coupling strengths compared to healthy controls (mean [SD]: −0.15 [0.11] vs. −0.22 [0.09], adjusted *p* = 0.013, Student's *t*‐test) (Figure 2D). The difference was also observed in the frontal (mean [SD]: −0.14 [0.11] vs. −0.21 [0.08], adjusted *p* = 0.045, Student's *t*‐test, FDR‐corrected) and temporal regions (−0.15 [0.11] vs. −0.22 [0.09], adjusted *p* = 0.045, Student's *t*‐test, FDR‐corrected) (Figure S1B).

**FIGURE 3:**
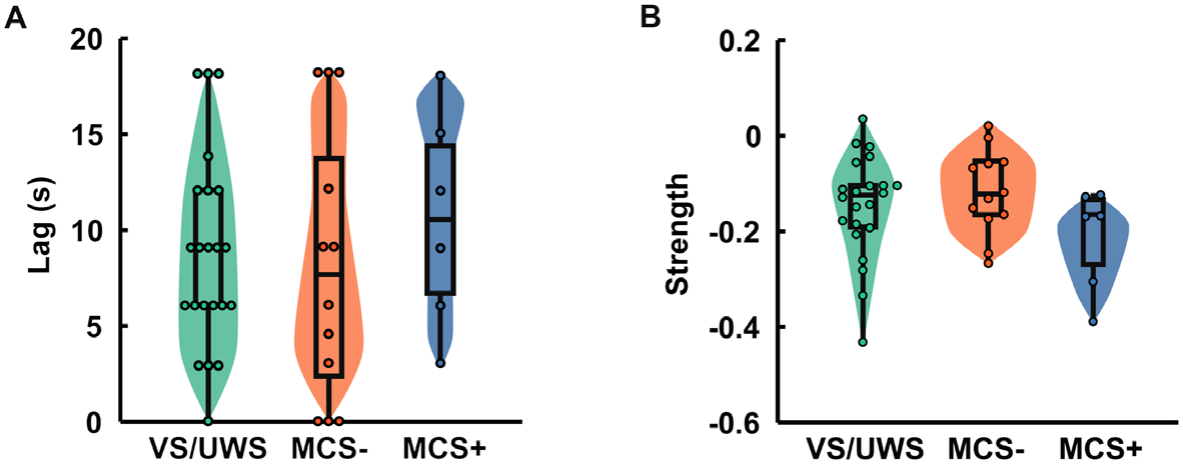
Comparisons of global BOLD‐CSF coupling time‐lag and strength in pDoC patients with varying baseline consciousness states. (A) Comparisons of global BOLD‐CSF coupling time‐lag across the different consciousness groups. (B) Comparisons of global BOLD‐CSF coupling strength across the different consciousness groups.

Patients with pDoC were then categorized into subgroups based on the etiologies. No significant differences were found in either time‐lags or strengths of global or regional BOLD‐CSF couplings between these subgroups (Figures S2 and S3). Similarly, no significant differences in time‐lags or strengths were observed among pDoC patients with different levels of consciousness (Figure 3, Figures S4 and S5).

We conducted correlation analyses between the clinical demographic data and the BOLD‐CSF coupling indicators and found no significant correlations between any of the clinical demographic variables and the BOLD‐CSF coupling indicators (Tables S2 and S3).

### 3.3 BOLD‐CSF Coupling in Patients With Different Prognosis

In the improved consciousness group, the mean BOLD‐CSF coupling resembled that of healthy controls, with a negative peak at a time‐lag of 3 s (*r* range: −0.008 to −0.104, *p* = 0.010, permutation test) (Figure 2E). In contrast, the unimproved consciousness group exhibited a distinct BOLD‐CSF coupling pattern, characterized by a significant negative peak at a time‐lag of 9 s (*r* range: −0.013 to −0.082, *p* = 0.021, permutation test) (Figure 2F).

Quantitative analysis revealed significantly prolonged global BOLD‐CSF coupling time‐lags in the unimproved consciousness group compared with the improved consciousness group (median [IQR]: 9.00 [9.00, 14.25] vs. 6.00 [3.00, 6.00], *p* < 0.001, Mann–Whitney *U* test) (Figure 2G). Similar increases were observed in regional BOLD‐CSF time‐lags, including the frontal (9.00 [6.75, 14.25] vs. 6.00 [3.00, 12.00], adjusted *p* = 0.030, Mann–Whitney *U* test, FDR‐corrected), parietal (10.50 [6.75, 17.25] vs. 3.00 [3.00, 6.00], adjusted *p* = 0.003, Mann–Whitney *U* test, FDR‐corrected), occipital (10.50 [6.75, 15.00] vs. 3.00 [0.00, 6.00], adjusted *p* = 0.005, Mann–Whitney *U* test, FDR‐corrected), and temporal (12.00 [9.00, 15.00] vs. 3.00 [0.00, 9.00], adjusted *p* = 0.018, Mann–Whitney *U* test, FDR‐corrected) regions (Figure S1C). However, no significant differences were found in BOLD‐CSF coupling strengths between the two outcome groups (Table 1, Figure 2H, Figure S1D).

### 3.4 Global BOLD‐CSF Time‐Lag Predicts Consciousness Recovery in Patients With pDoC

**FIGURE 4:**
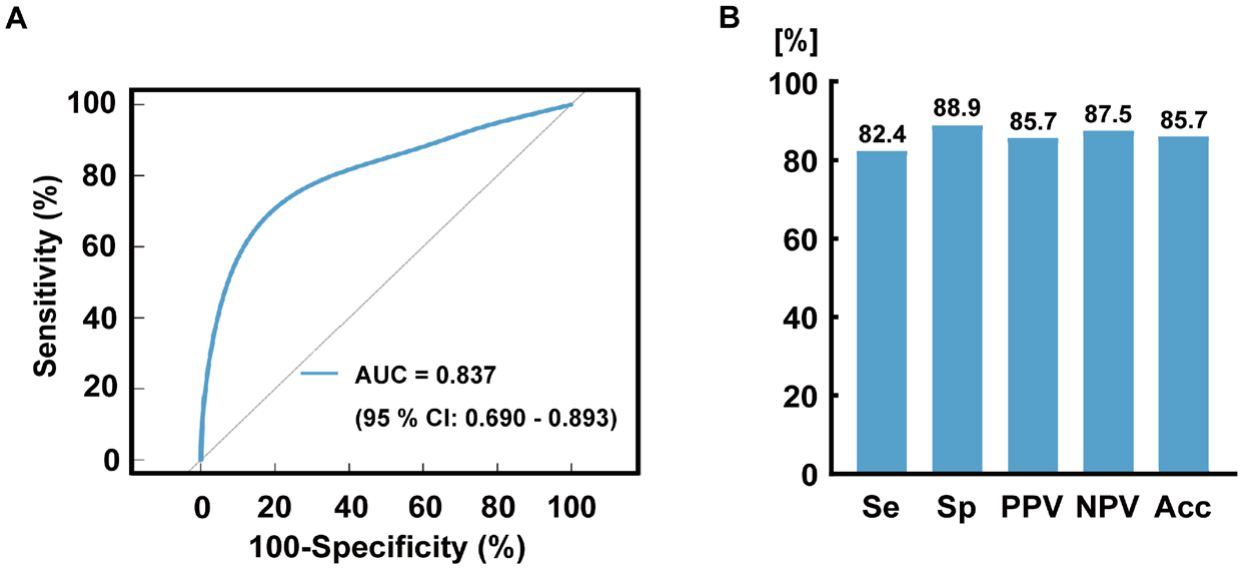
Predictive performance of global BOLD‐CSF coupling time‐lags for improved consciousness at 6 months. (A) Receiver operating characteristic curve of global BOLD‐CSF coupling time‐lags for predicting consciousness recovery in pDoC patients. (B) Sensitivity, specificity, positive predictive value, negative predictive value, and accuracy of global BOLD‐CSF coupling time‐lags for predicting consciousness recovery in pDoC patients. Acc, accuracy; AUC, area under the receiver operating characteristic curve; CI, confidence interval; NPV, negative predictive value; pDoC, prolonged disorders of consciousness; PPV, positive predictive value; Se, sensitivity; Sp, specificity.

We used bidirectional stepwise logistic regression to identify significant variables associated with the 6‐month prognosis of patients with pDoC. Our analysis revealed that global BOLD‐CSF time‐lag was the only variable significantly associated with patients' outcomes (*p* = 0.039, OR = 0.61, 95% CI: 0.38–0.98) (Table 2). Further evaluation of the predictive performance of the global BOLD‐CSF time‐lag regarding consciousness improvement in pDoC patients 6 months later demonstrated a good predictive performance, with an area under the curve (AUC) of 0.837 (95% CI: 0.690–0.980) (Figure 4A). Using a cut‐off value of 7.5 s, the global BOLD‐CSF time‐lag achieved a sensitivity of 82.4%, specificity of 88.9%, PPV of 85.7%, and NPV of 87.5% for predicting consciousness recovery. The overall predictive accuracy was 85.7% (Figure 4B).

**TABLE 2:** Multivariable logistic regression model for predicting consciousness recovery 6 months after enrollment.

BOLD‐CSF coupling time‐lags	Odds ratio (95% CI)	*p*
Global	0.61 (0.38 ~ 0.98)	**0.039**
Frontal	1.32 (0.94 ~ 1.87)	0.109
Occipital	0.80 (0.63 ~ 1.01)	0.060

*Note:* Statistically significant effect is marked in bold (*p* < 0.05).

Abbreviations: BOLD‐CSF coupling, the coupling between blood‐oxygen‐level‐dependent signals and cerebrospinal fluid signals; CI, confidence interval.

## 4 Discussion

In our study, we found that patients with pDoC had impaired glymphatic function, as was evidenced by delayed BOLD‐CSF coupling time‐lags and reduced coupling strengths. This suggested that the coupling between neural activity and CSF flow in patients with pDoC was abnormal. Moreover, the time‐lag in global BOLD‐CSF coupling serves as a prognostic biomarker of consciousness recovery after 6 months.

One pivotal finding of this study was that patients with pDoC had significant prolonged time‐lags in the BOLD‐CSF coupling, characterized by a delayed appearance of the negative correlation peak. To the best of our knowledge, this is the first report on the delay of BOLD‐CSF coupling. Neural activity triggers widespread cortical BOLD changes, which drive cerebral blood inflow and subsequent CSF exchange [36]. Recent research has further emphasized that neural oscillations serve as the primary driving force behind glymphatic perfusion [37]. The time‐lag of the negative peak in BOLD‐CSF correlation reflects the delay in the exchange between cerebral blood and CSF inflow, thereby indicating the efficiency of neural activity in recruiting CSF perfusion. In healthy controls, this time‐lag typically ranges from 2 to 4 s [38]. However, in patients with pDoC, we observed a significant prolongation of this time‐lag, extending to approximately 9 s. This delay suggested the impaired efficacy of neural activity in recruiting CSF inflow, and it may be linked to the disruption of neural oscillations in pDoC patients [20, 39].

In our analysis of BOLD‐CSF coupling strength, we found that in addition to global BOLD‐CSF coupling, the coupling strength in the frontal and temporal lobes of patients with pDoC was significantly lower compared with healthy controls. Previous studies in neurodegenerative diseases have shown that reduced BOLD‐CSF coupling strength is associated with worse cognitive and motor outcomes, such as greater cognitive decline in Alzheimer's disease and faster motor progression in Parkinson's disease [28, 40]. In our analysis of BOLD‐CSF coupling strength, we found that in addition to global BOLD‐CSF coupling, the coupling strength in the frontal and temporal lobes of patients with pDoC was significantly lower compared with healthy controls. This finding suggests a diminished capacity of the frontal and temporal lobes to recruit CSF in pDoC patients. In consistency, Panda et al. demonstrated that patients with pDoC exhibit a notable reduction in neural activity and network integration in the frontotemporal lobe, as inferred from rs‐fMRI data and 18F‐fluorodeoxyglucose‐PET scans [41]. These results not only suggest a critical role of frontoparietal dysfunction in the occurrence of DoC, but also indicate a close relationship between frontoparietal neural activity and the recruitment of glymphatic perfusion [41].

The recovery of consciousness remains a critical goal in the clinical management of pDoC [4]. Patients with pDoC experience a prolonged course, with changes in consciousness occurring very slowly, often requiring an extended period to demonstrate even minimal improvement. Therefore, the ability to predict consciousness recovery accurately and timely manner is of paramount importance. Accurate and timely prognostic assessments can significantly aid treatment decisions, facilitate the selection of appropriate therapeutic approaches, improve patient outcomes, and reduce unnecessary waste of medical resources [4, 6]. In our study, we found that the global BOLD‐CSF coupling time‐lag in pDoC patients effectively predicted consciousness improvement at the 6‐month follow‐up and demonstrated robust predictive performance (AUC = 0.837). This indicates that BOLD‐CSF coupling time‐lag is a good prognostic tool for pDoC. We surmise that the global BOLD‐CSF coupling time‐lag reflects the overall structural and functional integrity of the brain's glymphatic system [42]. A normal time‐lag indicates relatively preserved glymphatic function, promoting efficient clearance of toxic metabolites and waste, thus supporting better recovery of consciousness and predicting improved outcomes [43]. Notably, while BOLD‐CSF coupling time‐lag demonstrated prognostic value for long‐term consciousness recovery, it did not differentiate baseline consciousness states, suggesting its utility lies in predicting neuroplastic potential rather than diagnostic classification.

This study had several limitations. First, the enrolled patients had heterogeneous etiologies. Although this reflects the real‐world situation of pDoC patients, patients with different etiologies may exhibit different features of glymphatic impairment. To account for the impact of these different etiologies on BOLD‐CSF indicators, subgroup analyses were performed, revealing similar strengths and time lags of impairment across various causes. This suggests that the coupling impairment between neural activity and CSF inflow is a general characteristic of pDoC, rather than specific to any one etiology. Secondly, the use of different MRI machines to acquire imaging data introduced heterogeneity. We prioritized PET/MRI in data collection for pDoC patients due to its clinical relevance, but ethical concerns prevented its use on healthy individuals. To address this imaging heterogeneity, we applied the same fMRI acquisition sequence to standardize differences [25]. Thirdly, the study's relatively small sample size and short follow‐up period may limit the generalizability of the findings. While the global BOLD‐CSF coupling time lag showed strong predictive performance for consciousness recovery, it does not distinguish between different states of consciousness. Future efforts should focus on increasing the sample size and extending the follow‐up period to enhance the applicability of the results. Lastly, the absence of follow‐up MRI scans in our study precluded analysis of longitudinal BOLD‐CSF signal changes and their potential association with consciousness recovery. Future studies incorporating serial neuroimaging assessments are needed to characterize the dynamic relationship between BOLD‐CSF coupling and recovery trajectories in pDoC patients.

## 5 Conclusion

This study reveals impaired glymphatic function in patients with pDoC, as indicated by prolonged BOLD‐CSF coupling time‐lags and reduced coupling strengths. We also found that global BOLD‐CSF coupling time‐lags may serve as a prognostic biomarker for consciousness recovery. By linking neural activity, glymphatic function, and clinical outcomes, this research highlights the importance of glymphatic function in the recovery of consciousness in pDoC.

## Author Contributions

Dian‐Wei Wu, Chang‐Geng Song, Qiong Gao, and Wen Jiang contributed to the conception and design of the study. Dian‐Wei Wu, Rong Chen, Jing‐Jing Zhao, and Xuan Wang contributed to the acquisition and analysis of data. Dian‐Wei Wu, Chang‐Geng Song, Jing‐Jing Zhao, Ying‐Chi Zhang, Xiao‐Gang Kang, Qiong Gao, and Wen Jiang contributed to drafting the text or preparing the figures. Dian‐Wei Wu and Chang‐Geng Song contributed equally to this work as the co‐first authors. Qiong Gao and Wen Jiang contributed equally to this work as co‐corresponding authors.

## Conflicts of Interest

The authors declare no conflicts of interest.

## Supporting information


Data S1:

## Data Availability

The data that support the findings of this study are available on request from the corresponding author. The data are not publicly available due to privacy or ethical restrictions.
