# Supplementary material for: Glymphatic Function as a Prognostic Biomarker in Prolonged Disorders of Consciousness
Source: CNS Neurosci Ther. 2025 Jul 23;31(7):e70526. doi: 10.1111/cns.70526 (PMC12287379; doi:10.1111/cns.70526)
Supplement: Supplementary file 1 — Data S1: [file CNS-31-e70526-s001.docx]

**Supplementary Materials for:**

**Glymphatic Function as a Prognostic Biomarker in Prolonged**

**Disorders of Consciousness**

Dian-Wei Wu^1,†^, Chang-Geng Song^1,†^, Rong Chen^1^, Jing-Jing Zhao^1^, Ying-Chi Zhang^1^, Xuan Wang^1^, Zhong-Qing Sun^1^, Xiao-Gang Kang^1^, Qiong Gao^1,^* and Wen Jiang^1,^*

**^†^ Dian-Wei Wu and Chang-Geng Song contributed equally to this work.**

*** Corresponding authors**

**Author affiliation:**

^1^ Department of Neurology, Xijing Hospital, Fourth Military Medical University, Xi’an, 710032, China.

*** Correspondence to:**

Wen Jiang, Department of Neurology, Xijing Hospital, Fourth Military Medical University, Xi’an, 710032, China; Email: jiangwen@fmmu.edu.cn; Tel: +86-29-84771319.

Qiong Gao, Department of Neurology, Xijing Hospital, Fourth Military Medical University, Xi’an, 710032, China; Email: gaoqiong123@fmmu.edu.cn; Tel: +86-29-84771319.

| **Contents** |
| --- |
| **Table S1.** Demographic and clinical characteristics of all participants. |
| **Table S2.** Correlation between clinical demographic data and BOLD-CSF coupling time-lags. |
| **Table S3.** Correlation between clinical demographic data and BOLD-CSF coupling strengths. |
| **Figure S1.** Comparisons of regional BOLD-CSF coupling indicators in healthy controls and pDoC patients. |
| **Figure S2.** Comparisons of global and regional BOLD-CSF coupling time-lags in pDoC patients with different etiologies. |
| **Figure S3.** Comparisons of global and regional BOLD-CSF coupling strengths in pDoC patients with different etiologies. |
| **Figure S4.** Comparisons of global and regional BOLD-CSF coupling time-lags in pDoC patients with varying baseline consciousness states. |
| **Figure S5.** Comparisons of global and regional BOLD-CSF coupling strengths in pDoC patients with varying baseline consciousness states. |

**Table S1.** Demographic and clinical characteristics of all participants.

| **Participants** | **Age (years)** | **Gender** | **Etiology** | **Time since injury (days)** | **Consciousness** | **Baseline**  **CRS-R** | **Neuroimage** | **Outcome** |
| --- | --- | --- | --- | --- | --- | --- | --- | --- |
| Patient 1 | 22 | Male | Anoxia | 250 | MCS plus | 13 | Diffuse brain swelling | MCS plus |
| Patient 2 | 55 | Male | Metabolic encephalopathy | 29 | MCS minus | 12 | Demyelination in bilateral periventricular areas | MCS plus |
| Patient 3 | 35 | Male | Anoxia | 60 | VS/UWS | 3 | Brain edema and cortical swelling | VS/UWS |
| Patient 4 | 40 | Female | Anoxia | 29 | VS/UWS | 6 | Ischemic changes in cortices of bilateral cerebral  hemispheres | MCS minus |
| Patient 5 | 21 | Male | Anoxia | 98 | MCS minus | 12 | Diffuse ischemic changes in the left parietal lobe, bilateral insular lobes, basal ganglia, and cerebellar hemispheres | MCS minus |
| Patient 6 | 40 | Male | Anoxia | 58 | VS/UWS | 4 | Demyelination in subcortices of bilateral frontal and parietal lobes | MCS minus |
| Patient 7 | 53 | Female | Cerebral hemorrhage | 86 | MCS minus | 7 | Interstitial cerebral edema and demyelination in bilateral periventricular areas | MCS plus |
| Patient 8 | 43 | Male | Traumatic brain injury | 511 | MCS plus | 15 | Softening in the left lateral ventricle and right basal ganglia | MCS plus |
| Patient 9 | 46 | Female | Cerebral hemorrhage | 103 | MCS minus | 9 | Hemorrhage in the right thalamus | MCS plus |
| Patient 10 | 67 | Male | Cerebral infarction | 46 | MCS minus | 9 | Infarction in the right occipital lobe | MCS plus |
| Patient 11 | 64 | Female | Metabolic encephalopathy | 29 | VS/UWS | 6 | Osmotic demyelination in bilateral caudate nuclei, lentiform nuclei, thalamus, and brainstem | VS/UWS |
| Patient 12 | 61 | Male | Traumatic brain injury | 67 | MCS minus | 9 | Contusion and laceration in the left frontal lobe and bilateral temporal lobes | EMCS |
| Patient 13 | 34 | Male | Anoxia | 208 | VS/UWS | 7 | Ischemic changes in cortices of bilateral frontal and temporal lobes, as well as the basal ganglia | MCS minus |
| Patient 14 | 41 | Male | Metabolic encephalopathy | 44 | MCS minus | 12 | Osmotic demyelinationin bilateral temporal lobes, hippocampus and lentiform nucleus | MCS minus |
| Patient 15 | 21 | Male | Anoxia | 58 | VS/UWS | 6 | Demyelination in the right centrum semiovale | MCS minus |
| Patient 16 | 46 | Male | Anoxia | 57 | MCS minus | 10 | Demyelination in subcortices of bilateral frontal and parietal lobes | MCS plus |
| Patient 17 | 66 | Male | Cerebral hemorrhage | 34 | VS/UWS | 3 | Hemorrhage in the left frontal, parietal, temporal, occipital lobes, and basal ganglia | VS/UWS |
| Patient 18 | 48 | Female | Cerebral hemorrhage | 83 | MCS plus | 16 | Hemorrhage in the left thalamus | MCS plus |
| Patient 19 | 60 | Female | Traumatic brain injury | 32 | MCS minus | 7 | Contusion and laceration of the left temporal lobe and frontal lobe | MCS minus |
| Patient 20 | 60 | Female | Cerebral infarction | 74 | MCS plus | 14 | Multiple demyelination in the bilateral frontal, parietal, and temporal lobes, bilateral centrum semiovale, and bilateral periventricular regions | MCS plus |
| Patient 21 | 58 | Female | Cerebral hemorrhage | 29 | MCS minus | 8 | Hemorrhage in the brainstem | MCS plus |
| Patient 22 | 47 | Female | Anoxia | 145 | VS/UWS | 4 | Extensive ischemic changes in bilateral cerebral hemispheres, cerebral atrophy | VS/UWS |
| Patient 23 | 50 | Male | Cerebral hemorrhage | 48 | MCS plus | 15 | Hemorrhage in the left frontal, parietal, and temporal lobes, as well as basal ganglia and periventricular regions | EMCS |
| Patient 24 | 47 | Female | Anoxia | 60 | VS/UWS | 6 | Diffuse brain swelling | VS/UWS |
| Patient 25 | 56 | Male | Anoxia | 40 | VS/UWS | 8 | Ischemic changes in the bilateral basal ganglia and periventricular regions | VS/UWS |
| Patient 26 | 36 | Male | Traumatic brain injury | 29 | VS/UWS | 2 | Cerebral contusion and laceration in the left frontal lobe and temporal lobe | MCS minus |
| Patient 27 | 38 | Male | Anoxia | 29 | VS/UWS | 5 | Ischemic changes in the bilateral periventricular regions and centrum semiovale | VS/UWS |
| Patient 28 | 60 | Male | Traumatic brain injury | 56 | MCS plus | 17 | Contusion and laceration of the bilateral frontal lobes and corpus callosum | EMCS |
| Patient 29 | 48 | Male | Anoxia | 49 | MCS minus | 10 | Hemorrhage in the right thalamus | MCS minus |
| Patient 30 | 65 | Female | Anoxia | 29 | VS/UWS | 4 | Demyelination in bilateral periventricular regions | VS/UWS |
| Patient 31 | 31 | Female | Anoxia | 60 | VS/UWS | 4 | Brain edema and cortical swelling | VS/UWS |
| Patient 32 | 34 | Female | Traumatic brain injury | 60 | VS/UWS | 8 | Contusion and laceration of the left temporal lobe and bilateral periventricular regions | MCS plus |
| Patient 33 | 60 | Female | Metabolic encephalopathy | 29 | MCS minus | 10 | Demyelination in bilateral periventricular regions | EMCS |
| Patient 34 | 71 | Male | Anoxia | 49 | VS/UWS | 4 | Demyelination in bilateral centrum semiovale and periventricular regions | VS/UWS |
| Patient 35 | 45 | Female | Anoxia | 45 | VS/UWS | 5 | Multiple softening in the brainstem, right frontal and temporal lobes, periventricular areas, basal ganglia, and thalamus | MCS minus |
| Patient 36 | 31 | Male | Cerebral infarction | 127 | VS/UWS | 7 | Softening in the pons and left cerebellum | MCS  minus |
| Patient 37 | 52 | Male | Anoxia | 45 | VS/UWS | 5 | Multiple softening with surrounding gliosis in the bilateral centrum semiovale, periventricular areas, right basal ganglia, and brainstem | VS/UWS |
| Patient 38 | 28 | Female | Anoxia | 29 | VS/UWS | 3 | DWI hyperintense lesions in cortices of bilateral temporal and occipital lodes, bilateral caudate nuclei, and lentiform nuclei | VS/UWS |
| Patient 39 | 57 | Male | Cerebral infarction | 90 | VS/UWS | 6 | Cortical atrophy in the left frontal, temporal, parietal, and occipital lobes | MCS minus |
| Patient 40 | 65 | Male | Anoxia | 65 | VS/UWS | 4 | Ischemic changes in bilateral basal ganglia and white matter of the bilateral frontal lobes, cerebral atrophy | VS/UWS |
| Healthy control 1 | 30 | Male | - | - | - | - | - | - |
| Healthy control 2 | 35 | Female | - | - | - | - | - | - |
| Healthy control 3 | 53 | Female | - | - | - | - | - | - |
| Healthy control 4 | 29 | Male | - | - | - | - | - | - |
| Healthy control 5 | 34 | Male | - | - | - | - | - | - |
| Healthy control 6 | 39 | Female | - | - | - | - | - | - |
| Healthy control 7 | 35 | Male | - | - | - | - | - | - |
| Healthy control 8 | 42 | Male | - | - | - | - | - | - |
| Healthy control 9 | 31 | Male | - | - | - | - | - | - |
| Healthy control 10 | 49 | Male | - | - | - | - | - | - |
| Healthy control 11 | 48 | Female | - | - | - | - | - | - |
| Healthy control 12 | 31 | Male | - | - | - | - | - | - |
| Healthy control 13 | 47 | Male | - | - | - | - | - | - |
| Healthy control 14 | 56 | Female | - | - | - | - | - | - |
| Healthy control 15 | 38 | Female | - | - | - | - | - | - |
| Healthy control 16 | 41 | Female | - | - | - | - | - | - |
| Healthy control 17 | 58 | Male | - | - | - |  | - | - |
| Healthy control 18 | 43 | Male | - | - | - |  | - | - |
| Healthy control 19 | 37 | Male | - | - | - |  | - | - |
| Healthy control 20 | 62 | Male | - | - | - |  | - | - |

Abbreviations: CRS-R, coma recovery scale-revised; CT, computed tomography; DWI, diffusion weighted imaging; EMCS, emergence from minimally conscious state; MCS, minimally conscious state; VS/UWS, vegetative state/unresponsive wakefulness syndrome.

**Table S2**. Correlation between clinical demographic data and BOLD-CSF coupling time-lags

|  | **Global** | **Frontal** | **Parietal** | **Occipital** | **Temporal** | **Subcortex** |
| --- | --- | --- | --- | --- | --- | --- |
| Age | -0.121 (0.456) | -0.162 (0.317) | 0.116 (0.477) | -0.211 (0.191) | -0.126 (0.439) | -0.076 (0.641) |
| Sex | 0.085 (0.602) | -0.116 (0.476) | 0.103 (0.527) | -0.203 (0.209) | -0.009 (0.956) | -0.047 (0.773) |
| Time since injury | 0.240 (0.136) | 0.192 (0.236) | 0.092 (0.574) | 0.307 (0.054) | 0.199 (0.218) | 0.118 (0.470) |
| CRS-R | -0.115 (0.480) | -0.160 (0.325) | -0.051 (0.753) | -0.216 (0.181) | -0.168 (0.302) | -0.206 (0.203) |

**Note:** Values are shown as correlation coefficient (*p*-value). No significant result was found.

**Table S3** Correlation between clinical demographic data and BOLD-CSF coupling strengths

|  | **Global** | **Frontal** | **Parietal** | **Occipital** | **Temporal** | **Subcortex** |
| --- | --- | --- | --- | --- | --- | --- |
| Age | -0.125 (0.441) | -0.212 (0.190) | -0.037 (0.821) | -0.190 (0.240) | -0.177(0.275) | -0.144(0.376) |
| Sex | 0.217 (0.179) | -0.031 (0.850) | 0.181 (0.263) | -0.049 (0.766) | 0.221 (0.171) | 0.040 (0.807) |
| Time since injury | 0.087 (0.591) | 0.075 (0.645) | -0.071 (0.662) | -0.122 (0.455) | 0.231 (0.152) | 0.207 (0.200) |
| CRS-R | -0.129 (0.429) | 0.132 (0.416) | 0.087 (0.592) | -0.049 (0.763) | 0.059 (0.719) | 0.059 (0.719) |

**Note:** Values are shown as correlation coefficient (*p*-value). No significant result was found.

**
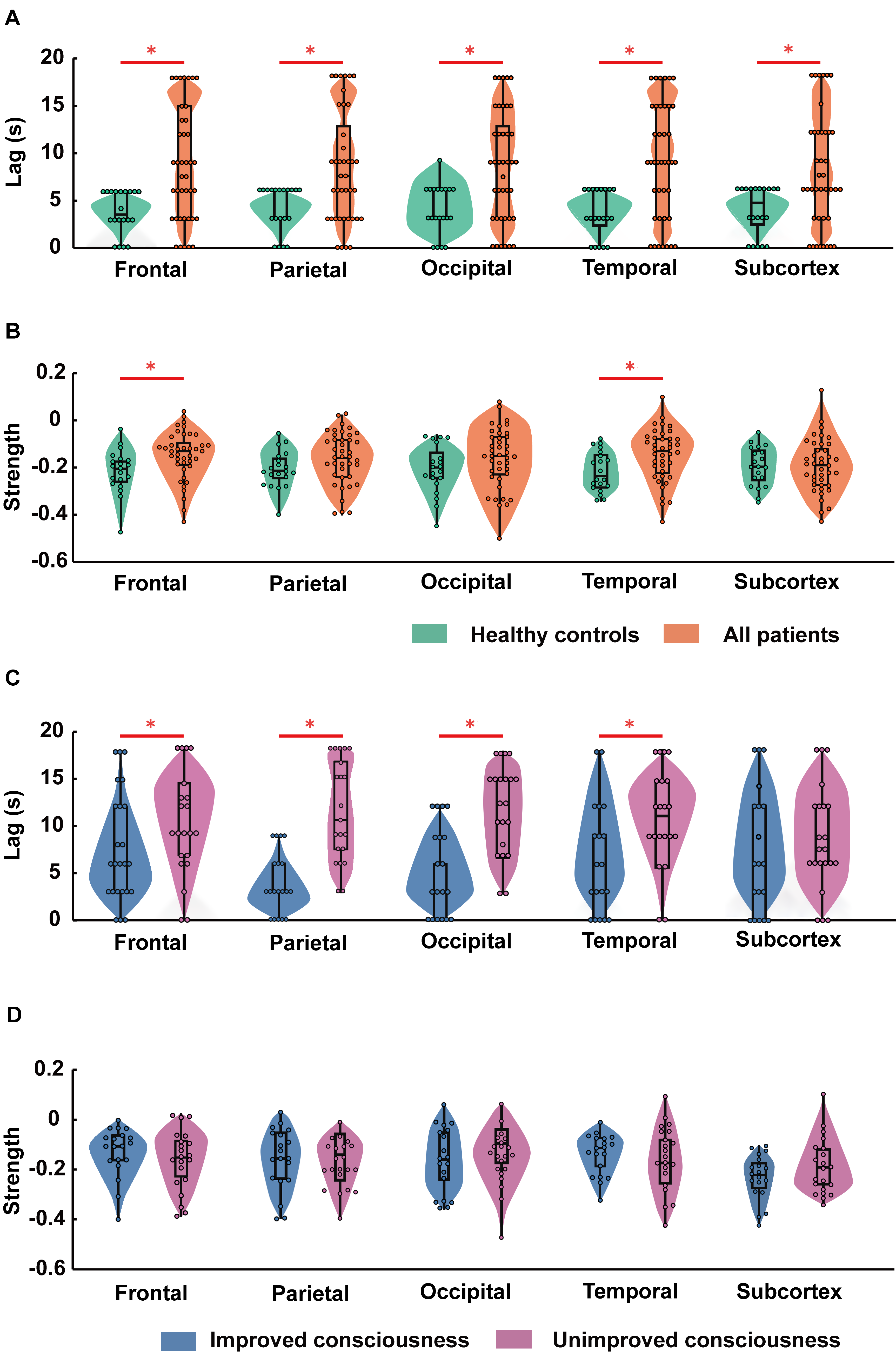
**

**Figure S1. Comparisons of regional BOLD-CSF coupling indicators in healthy controls and pDoC patients. (A)** Comparisons of regional BOLD-CSF coupling time-lags between healthy controls and all patients; **(B)** Comparisons of regional BOLD-CSF coupling strengths between healthy controls and all patients; **(C)** Comparisons of regional BOLD-CSF coupling time-lags between the improved and unimproved consciousness groups; **(D)** Comparisons of regional BOLD-CSF coupling strengths between the improved and unimproved consciousness groups. **p* < 0.05. The *p-*values were corrected using false discover rate correction.

**
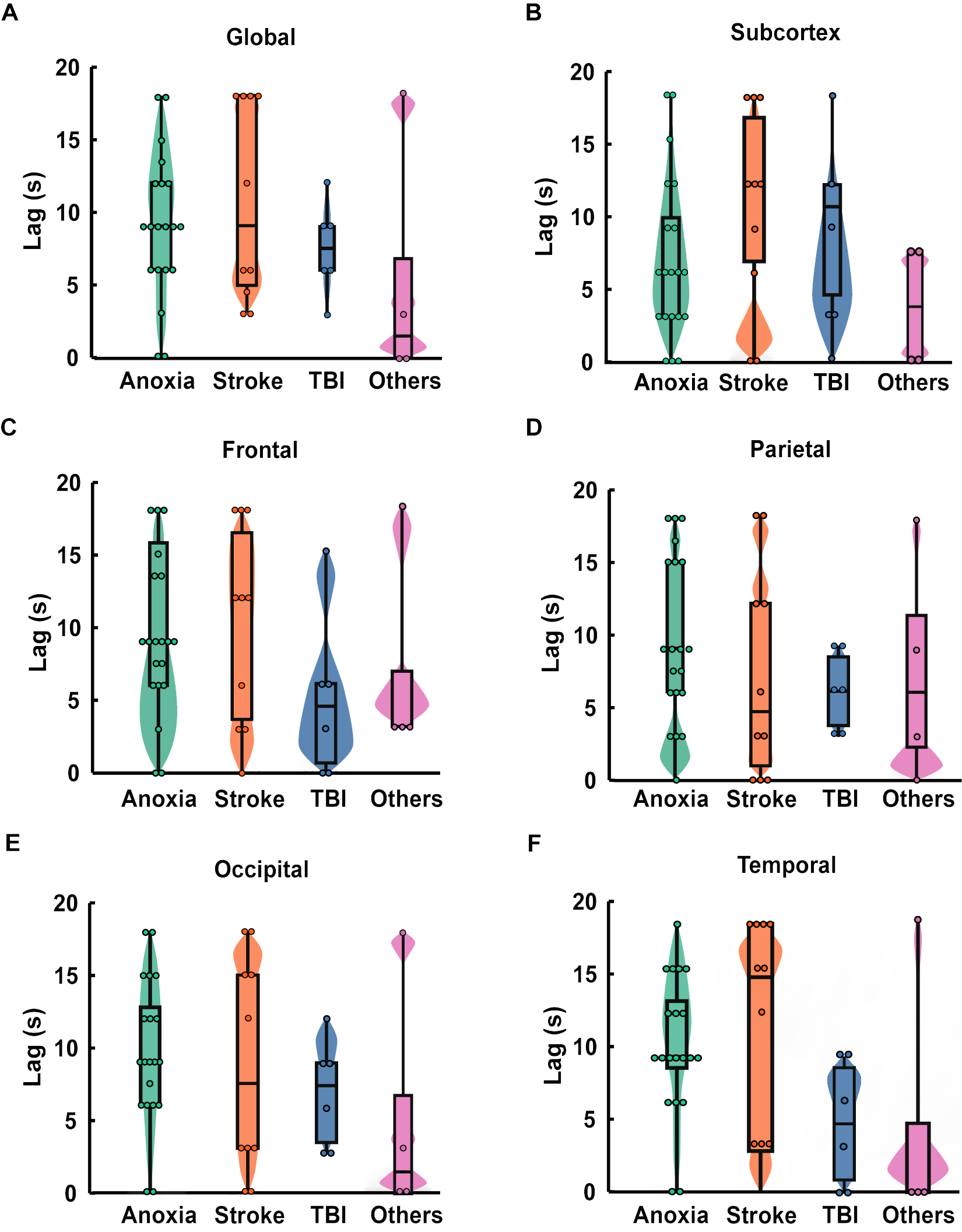
**

**Figure S2. Comparisons of global and regional BOLD-CSF coupling time-lags in pDoC patients with** **different etiologies.** **(A)** Comparisons of global BOLD-CSF coupling time-lags across the different etiological groups; **(B)** Comparisons of subcortex BOLD-CSF coupling time-lags across the different etiological groups; **(C)** Comparisons of frontal BOLD-CSF coupling time-lags across the different etiological groups; **(D)** Comparisons of parietal BOLD-CSF coupling time-lags across the different etiological groups; **(E)** Comparisons of occipital BOLD-CSF coupling time-lags across the different etiological groups; and **(F)** Comparisons of temporal BOLD-CSF coupling time-lags across the different etiological groups. No significant result was found. Abbreviations: TBI, traumatic brain injury.


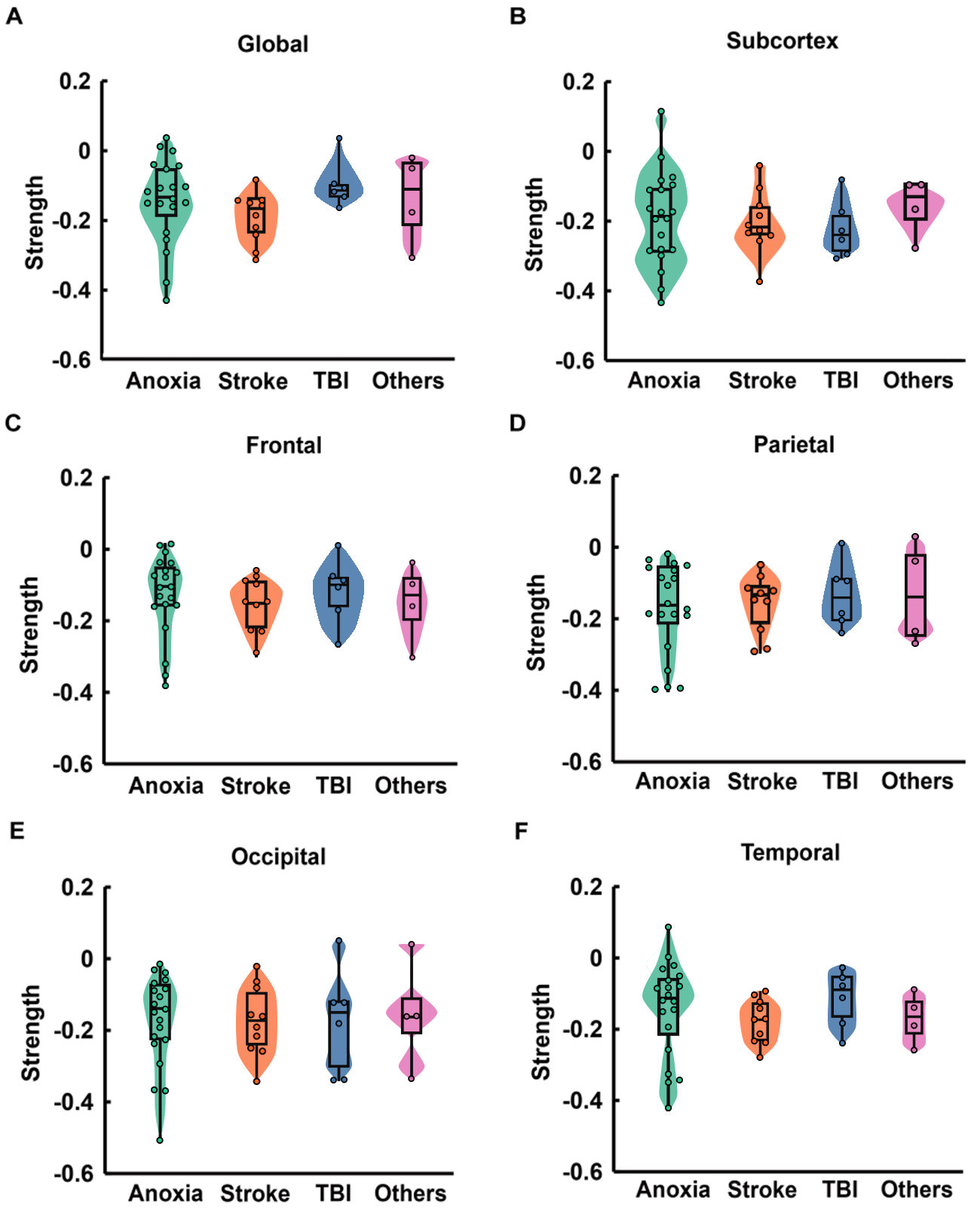


**Figure S3. Comparisons of global and regional BOLD-CSF coupling strengths in pDoC patients with different etiologies. (A)** Comparisons of global BOLD-CSF coupling strengths across the different etiological groups; **(B)** Comparisons of subcortex BOLD-CSF coupling strengths across the different etiological groups; **(C)** Comparisons of frontal BOLD-CSF coupling strengths across the different etiological groups; **(D)** Comparisons of parietal BOLD-CSF coupling strengths across the different etiological groups; **(E)** Comparisons of occipital BOLD-CSF coupling strengths across the different etiological groups; and **(F)** Comparisons of temporal BOLD-CSF coupling strengths across the different etiological groups. No significant result was found. Abbreviations: TBI, traumatic brain injury.

**
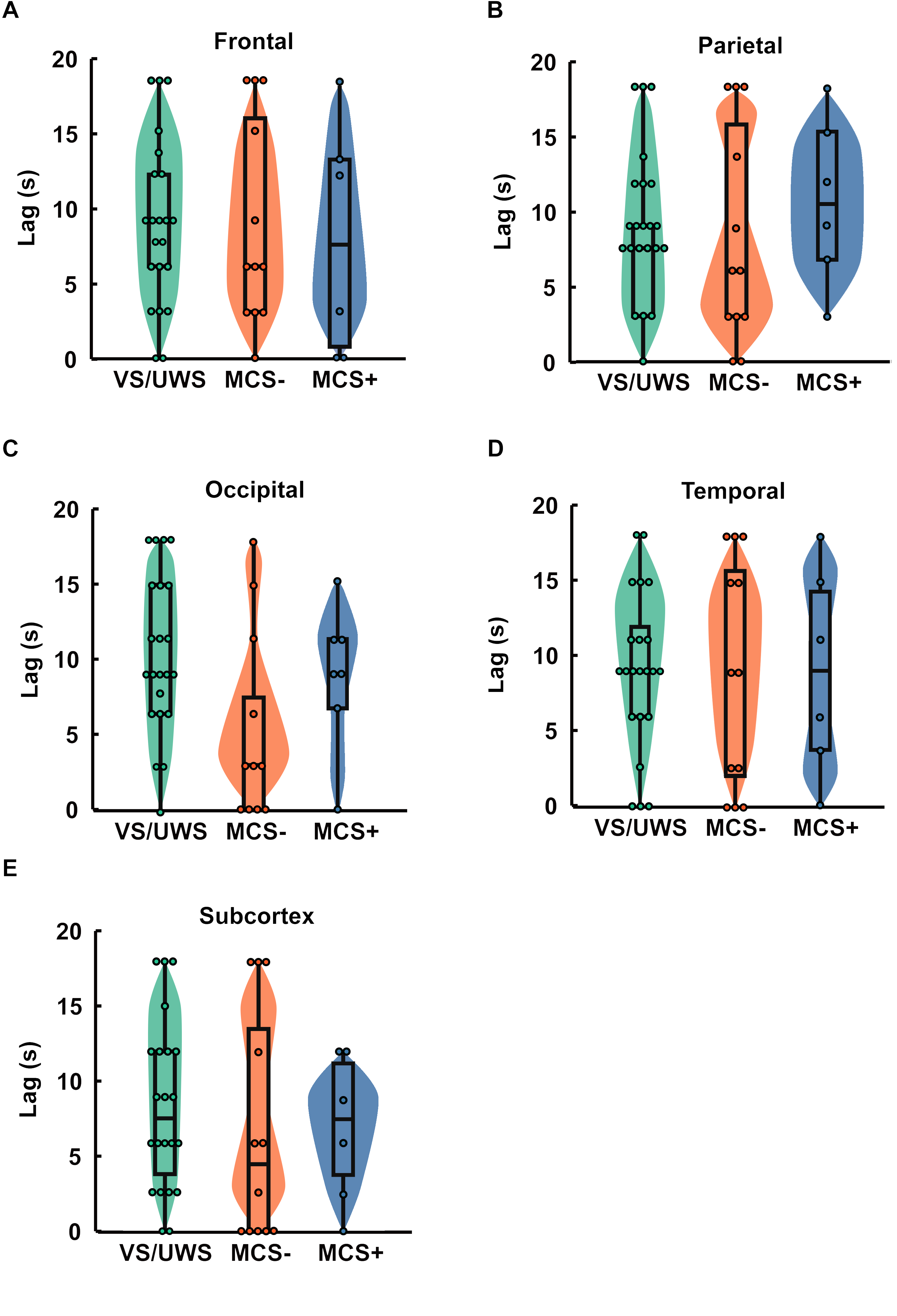
**

**Figure S4. Comparisons of regional BOLD-CSF coupling time-lags in pDoC patients with varying baseline consciousness states. (A)** Comparisons of frontal BOLD-CSF coupling time-lags across the different consciousness groups; **(B)** Comparisons of parietal BOLD-CSF coupling time-lags across the different consciousness groups; **(C)** Comparisons of occipital BOLD-CSF coupling time-lags across the different consciousness groups; **(D)** Comparisons of temporal BOLD-CSF coupling time-lags across the different consciousness groups; and **(E)** Comparisons of subcortex BOLD-CSF coupling time-lags across the different consciousness groups. No significant result was found. Abbreviations: MCS, minimally conscious state; VS/UWS, vegetative state/unresponsive wakefulness syndrome.

**
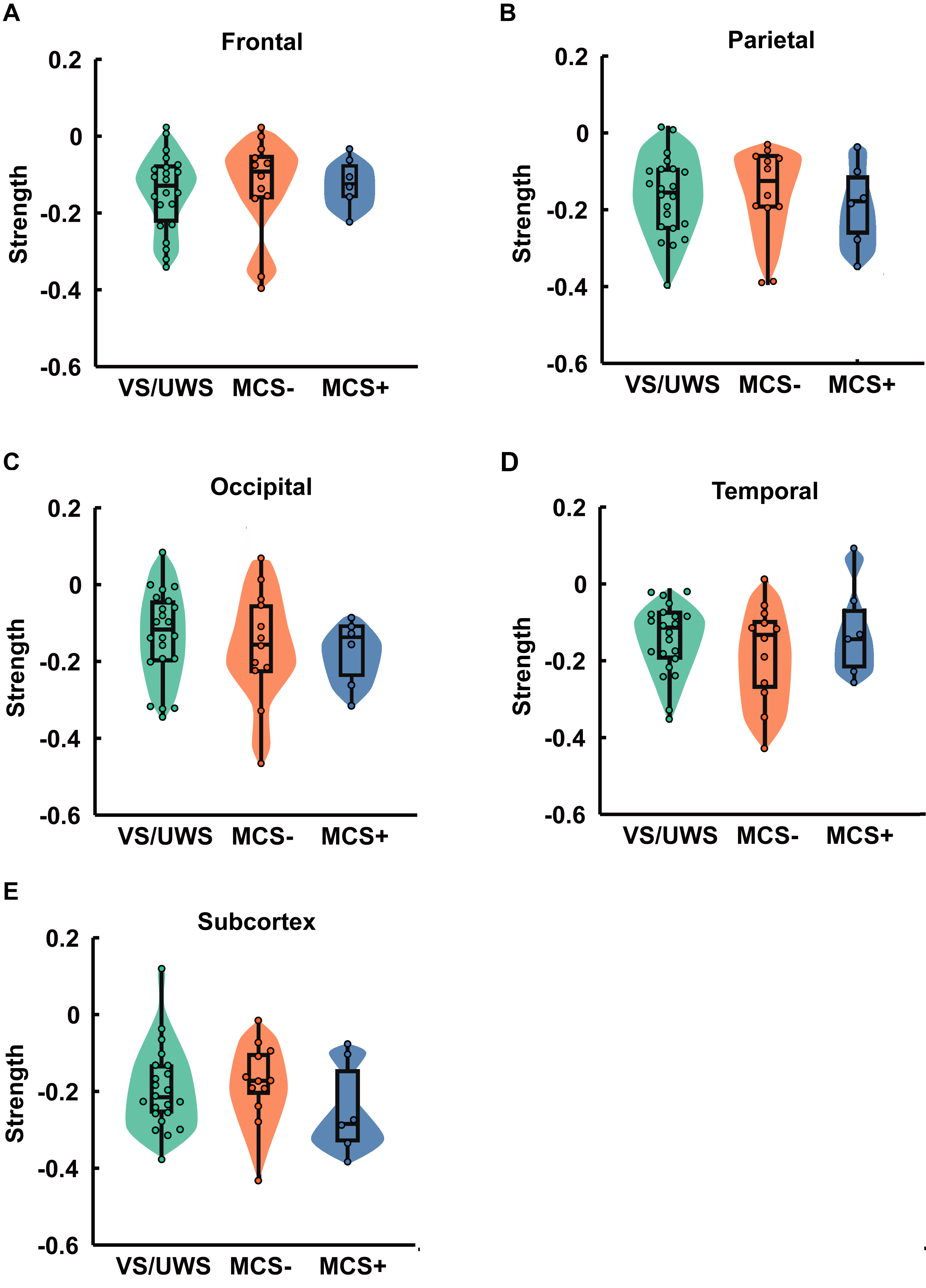
**

**Figure S5. Comparisons of regional BOLD-CSF coupling strengths in pDoC patients with varying baseline consciousness states. (A)** Comparisons of frontal BOLD-CSF coupling strengths across the different consciousness groups; **(B)** Comparisons of parietal BOLD-CSF coupling strengths across the different consciousness groups; **(C)** Comparisons of occipital BOLD-CSF coupling strengths across the different consciousness groups; **(D)** Comparisons of temporal BOLD-CSF coupling strengths across the different consciousness groups; and **(E)** Comparisons of subcortex BOLD-CSF coupling strengths across the different consciousness groups. No significant result was found. Abbreviations: MCS, minimally conscious state; VS/UWS, vegetative state/unresponsive wakefulness syndrome.
